# Supplementary material for: A deep learning approach to predict visual field using optical coherence tomography
Source: PLoS One. 2020 Jul 6;15(7):e0234902. doi: 10.1371/journal.pone.0234902 (PMC7337305; doi:10.1371/journal.pone.0234902)
Supplement: Attachment — Submitted filename: Response To Reviewers.docx [file pone.0234902.s002.docx]

Reviewer #1:

The paper is well-written, easy-to-understand and ordered reasonably. The statistical methods are correct and reliable and deep learning method is easy but accurate enough. My main criticism is about literature review which lacks in deep learning section, but mostly explores different methods for estimating VF using OCT information.

**Comment 1)** A very similar paper which should be explored thoroughly is: Deep Learning Approaches Predict Glaucomatous Visual Field Damage from OCT Optic Nerve Head En Face Images and Retinal Nerve Fiber Layer Thickness Maps by Mark Christopher. The authors should provide explanation on similarities and differences of their method compared to this work. They should also compare the results with their outcomes. It is also essential to site more papers about similar applications using deep learning to show the variety of possible application with deep learning, among which one may consider estimation of VF values.

🡺 Thank you for comment. We submitted this manuscript on 18^th^ Sep. 2019. It was before the Mark Christopher’s paper published (30^th^ Sep. 2019). So, we never saw Mark Christopher’s paper at the time we write our manuscript. However, it was surprising that our manuscript is very similar to Mark Christopher’s paper which we never saw before. We carefully read Mark Christopher’s paper and found similarities and differences. Major difference was that Mark Christopher’s method predicts only visual field global indices and some sectoral average pattern deviation values while our method predicts an entire picture of visual field. The backbone architecture was different in that they used only ONH image as input while our architecture takes both Macula and ONH images together. Christopher’s method used ResNet but our method uses Inception V3. We added sentences describing these differences in discussion section like below.

“In a recent study similar to ours, Christopher et al. [41] used a deep learning method to predict glaucomatous visual fields from OCT images. The deep learning architecture employed to predict visual field global indices was ResNet and the inputs were Spectralis SD-OCT ONH images. Various image types (RNFL thickness maps, RNFL en-face images, and confocal scanning laser ophthalmoscopic images) were input and the predictions using each type were compared. Unlike our deep learning method, which predicts the entire visual field from both macular and ONH images, the method of Christopher et al. uses only ONH image as input and predicts visual field global indices including the mean deviation (MD), the pattern standard deviation (PSD), and the mean sectoral pattern deviation. The best mean absolute errors, between the real and predicted values, were 2.5 dB (MD) and 1.5 dB (PSD).”

- The English in this document has been checked by at least two professional editors, both native speakers of English. For a certificate, please see: http://www.textcheck.com/certificate/GIKtlz

**Comment 2)** The second issue is regarding the proposed deep learning structure which needs some more explanation. Loss functions and activation maps in each layer, number of frozen layers in fine tuning stage, and comparison with other possible network structures should be elaborated in more detail. Furthermore, depicting some plots from learning curves may be informative to the reader.

🡺 Thank you for comment. We agree with you. First of all, we modified figure 1 which describes deep learning architecture. Please see below.

The major change was we added activation function (Relu) in dense layers. Inception V3 is an open source architecture and a lot of web sites explain the details of it. Thus, we did not include the inside structure of Inception V3. Instead, for readers who want to know more detailed information of architecture, we uploaded all our python source codes (VFOCT_Train.py, TestModel.py, VFOCT_Model.py, VFOCT_DataLoad.py), and train log files (learning curves) to Github. **Now, everybody can try our source code.** Please visit Github: <https://github.com/climyth/VFbySD-OCT>. And to give readers the details of training, we modified paragraph in method section like below (important changes are red colored).

<original paragraph in method section>

“Before training began, Inception V3 pretrained on the ImageNet dataset was downloaded and applied. Instead of transfer learning, we used fine tuning as OCT images are completely different images to those in the ImageNet dataset. Two thousand eight hundred and eleven training data records were randomly split into a training set and validation set at a ratio of 9:1, and a batch size of 64 records were supplied to the neural network. The training process was monitored using the accuracy trend of both the training and validation sets. When no more accuracy gains were observed and the validation accuracy began to decrease while training accuracy continued to increase (i.e. the neural network was being overfitted), training was ended.”

<modified version. Important changes are red colored>

“Before training began, Inception V3 pretrained on the ImageNet dataset was downloaded and applied. No layer was frozen during training and all layers were fine-tuned. A total of 2,811 records were randomly split into training and validation datasets in a 9:1 ratio and batches of 64 were supplied to the neural network. The optimizer was ‘rmsprop’ and the loss function was ‘mean squared error’. Training was monitored by reference to the loss trends of both the training and validation sets. When no further performance gain was observed over 100 epochs, training finished. To prevent overfitting, the repeated random sub-sampling cross validation technique [35] was used. The training data were again randomly split in a 9:1 ratio, the last trained weight file was loaded, and training resumed until no further performance gain was evident over 100 epochs. This process was repeated five times.”

- The English in this document has been checked by at least two professional editors, both native speakers of English. For a certificate, please see: http://www.textcheck.com/certificate/GIKtlz

**Comment 3)** Finally, the authors should provide more detailed explanation on production of class activation maps; namely, the information from maps in each regression output should be explained and compared with simple classification approaches.

🡺 Thank you for comment. We agree with you. Figure 4, CAM images, needs more detailed explanations with numbering. First, we modified the figure like below.

The major change is that

1) we added numbers in (C) to designate each CAM image in figure legend.
2) we added (D) to clarify structure-function mapping. This image shows the geometrical relationship between combined OCT image and visual field test points.

And we modified figure legend to explain more details of it like below (major changes are red colored).

**“Fig 4. A representative example of a class activation map (CAM).** The figure shows (A) the actual threshold values and (B) the predicted threshold values of the visual field examination. (C) Fifty-two CAMs were placed at individual visual field test points. Each CAM image is numbered at the top left. (D) Structure-function mapping between the combined OCT image (left) and the visual field (right). The macular scan in the combined OCT image corresponds to the dashed rectangle in the visual field. Color-coded Garway-Heath sectors are superimposed on the ONH scan of the combined OCT image and the corresponding visual field regions are similarly colored. The numbers in the visual field image are the same as those in the CAM images of (C).

In each CAM image, red indicates strongly activated points yielding high threshold values; blue (or no color) indicates the opposite. In this example, the visual field damage is principally superonasal; the inferonasal region is relatively intact (i.e., exhibits high threshold values). The CAM images in (C) that are numbered 27~30, 35~38, 43~46, and 49~52 are intensely red (high visual field threshold values). Note that these activated areas in the CAM images (also intensely red) exactly match the orange and dark grey regions in (D). In contrast, the CAM images numbered 5, 6, 11, 12, and 19~22 are not colored (and thus not activated) and generate low threshold visual field values. These areas match the blue and light grey regions in (D).”

- The English in this document has been checked by at least two professional editors, both native speakers of English. For a certificate, please see: http://www.textcheck.com/certificate/GIKtlz

Reviewer #2:

The authors present a deep learning approach in order to predict visual fields from optical coherence tomography (OCT) imaging. Based on the Google Inception V3 architecture, the authors trained their network on 1529 subjects (2811 eyes) and tested it on 290 subjects (290 eyes, 112 normal subjects and 178 glaucoma patients). Performance was evaluated using the RMSE, showing significant differences between normal subjects and patients with glaucoma (3.27 to 5.75). Importantly, the performance was also evaluated for different regions (Garway-Heath sectorisation and OCT scan area), in order to assess the regional specificity of the analysis. In addition, the authors perform many additional analyses with respect to the influence of different covariates including age, visual acuity, spherical equivalence etc. The study seems to be technically sound and conclusions are drawn appropriately based on the data presented. The discussion is comprehensive and covers related research. However, I would like to make the following comments:

**Comment 1)** What is the clinical significance of this study? Why should a deep learning network be used instead of an automated perimetry? In lines 414/415, the authors write that such a system could be useful for patients that are "unable to undergo an actual visual field exam". What would be the potential settings?

🡺 Thank you for comment. Visual field exam is a very import test to monitor glaucoma patients. However, in practice, it is also a very difficult test to perform. Most of all, patients need to pay attention for a long time. It takes about ten minutes per eye and 20 minutes per patient without rest. Many patients became tired during this boring test. Only 2~3 patients can take visual field exam per hour but many hospitals have only 1~2 Humphreys visual field machine(s). Moreover, the test itself is not easy. Patients require difficult learning curve. If patients are too young or too elder, they don’t learn how to do this exam. The reproducibility of visual field exam is not good. There is large amount of fluctuation. Please see the previous studies:

- Michael K. Birch, Determining Progressive Visual Field Loss in Serial Humphrey Visual Fields, Ophthalmology. 1995;102(8): 1227-1235
- Anders Heijil. Test-Retest Variability in Glaucomatous Visual Fields. Opthalmology. 19889;108 (2):130-135.

In contrast, OCT is relatively easy. It is just like a taking photo and requires only a few seconds. There is no learning curve in OCT exam. Thus, if we can predict visual field exam from OCT image, we expect the benefit will be a lot. Clinician can estimate visual field even in young children, people with mentally retarded and dementia. To clarify this, we modified last sentence of conclusion like below.

“In conclusion, the deep learning method effectively predicted the visual field 24-2 using the combined OCT image (mGCIPL and pRNFL thickness map) with a prediction error of 4.70 dB. The accuracy of the visual field prediction was not influenced by factors such as age, visual acuity, spherical equivalence, axial length, and OCT signal strength. This may help clinicians perform visual field testing, especially of patients who are unable to undergo real visual field examinations (young children, dementia patients, and mentally retarded patients).”

- The English in this document has been checked by at least two professional editors, both native speakers of English. For a certificate, please see: http://www.textcheck.com/certificate/GIKtlz

**Comment 2)** Some methodological details are missing on the way of fine-tuning and training process (lines 191-197): How did the authors perform the fine-tuning? And why is this opposed to transfer learning? Transfer learning can also be used in the framework of fine-tuning (e.g. weights of the pretrained network can be used as initialization for the new data, weights can be fixed in different layers). What kind of heuristic did the authors use for avoiding overfitting? Could the authors be a bit more specific about "When no more accuracy gains were observed..." (over a certain window size? How do the training and valdiation curve look like? How much variability?)?

🡺 Thank you for comment. We agree with you. As you said, transfer learning can be used in the framework of fine-tuning. We just wanted to emphasis that all layers of deep learning architecture were opened to training (no frozen layer) from the beginning of our training process. After first initialization of weight with ImageNet trained weights, our training data was randomly split at the ratio of 9:1 (train vs validation) and loss trend of both training set and validation set was monitored. No more performance gain was observed over 100 epochs or validation loss increases (early stop method) we stopped. To prevent overfitting, we used “repeated random sub-sampling cross validation” technique (also known as Monte-Carlo cross-validation) because it was simple to apply. Thus, after first training was over, the whole process was repeated for 5 times. The training data was randomly split at the ratio of 9:1 again and last weight file was loaded and then training was resumed until no more performance gain during about 100 epochs. We modified method section to include all these details like below.

<original paragraph in method section>

“Before training began, Inception V3 pretrained on the ImageNet dataset was downloaded and applied. Instead of transfer learning, we used fine tuning as OCT images are completely different images to those in the ImageNet dataset. Two thousand eight hundred and eleven training data records were randomly split into a training set and validation set at a ratio of 9:1, and a batch size of 64 records were supplied to the neural network. The training process was monitored using the accuracy trend of both the training and validation sets. When no more accuracy gains were observed and the validation accuracy began to decrease while training accuracy continued to increase (i.e. the neural network was being overfitted), training was ended.”

<modified version. Important changes are red colored>

“Before training began, Inception V3 pretrained on the ImageNet dataset was downloaded and applied. No layer was frozen during training and all layers were fine-tuned. A total of 2,811 records were randomly split into training and validation datasets in a 9:1 ratio and batches of 64 were supplied to the neural network. The optimizer was ‘rmsprop’ and the loss function was ‘mean squared error’. Training was monitored by reference to the loss trends of both the training and validation sets. When no further performance gain was observed over 100 epochs, training finished. To prevent overfitting, the repeated random sub-sampling cross validation technique [35] was used. The training data were again randomly split in a 9:1 ratio, the last trained weight file was loaded, and training resumed until no further performance gain was evident over 100 epochs. This process was repeated five times.”

- The English in this document has been checked by at least two professional editors, both native speakers of English. For a certificate, please see: http://www.textcheck.com/certificate/GIKtlz

**Comment 3)** No availability of code: Authors should make their code publicly available (e.g. via github) so that others can test the code on their data.

🡺 Thank you for your comment. We agree with you. We uploaded all our source code, final trained model file, and some test images on GitHub. Now, everyone can try our code. Please visit GitHub address below:

https://github.com/climyth/VFbySD-OCT

**Comment 4)** Availability of data: Authors state that the data is available on reasonable request. What do the authors mean by that? Are there any reasons (e.g. privacy, no consent) speaking against publishing the data?

🡺 Thank you for comment. Please understand that uploading medical images on the public storage is against the Korean law. Moreover, we trained our model with thousands of OCT images and if someone downloads it to use commercially, the legal problem may become bigger. IRB consent is also a problem. Even between Korean researchers, providing thousands of medical images to another hospital is not easy. Usually, hospital’s PACS departments are very reluctant to share their images with another hospital and we often failed to get consent from IRB too. Instead of uploading the whole image data, like answered in comment 3 above, we can upload all our source code and model as well as some depersonalized combined OCT images and visual field data for test purpose. We already provided all datasheet as supplementary file. As we wrote in manuscript already, we are willing to provide our OCT images on reasonable request (in this case, we must report to IRB and hospital. But if you need our data for research purpose, please do not hesitate to contact us).

**Comment 5)** The authors write in the abstract that they "developed a novel deep learning architecture", but the architecture is a variation of an existing architecture (Inception V3).

🡺 Thank you for comment. We agree with you. We have gone too far. It is more like a variation of Inception V3. We modified our abstract like below.

“We developed a novel deep learning architecture to predict visual field” 🡪 “We developed a deep learning architecture based on Inception V3 to predict visual field”

**Comment 6)** Why did the authors not evaluate the influence of sex in Table 4?

🡺 Thank you for comment. We added evaluation of sex in Table 4 and modified a sentence in result section like below. As you can see, Sex was not significantly associated with prediction error.

<Table 4>

|  | Correlation coefficients | | Simple linear regression analysis | | | |
| --- | --- | --- | --- | --- | --- | --- |
|  | Spearman’s rho | *P* value | Slope | Intercept | R^2^ | *P* value |
| Age | –0.003 | 0.957 | 0.011 | 4.230 | 0.004 | 0.301 |
| Sex | –0.018 | 0.756 | –0.108 | 4.842 | <0.001 | 0.722 |
| Visual acuity (logMAR) | 0.157 | 0.007 | 2.803 | 4.556 | 0.017 | 0.028 |
| Spherical equivalence | –0.018 | 0.765 | 0.015 | 4.818 | <0.001 | 0.766 |
| Central corneal thickness | –0.051 | 0.404 | –0.003 | 6.705 | 0.002 | 0.452 |
| Axial length | 0.071 | 0.257 | 0.015 | 4.399 | <0.001 | 0.878 |
| Visual field MD | –0.543 | <0.001 | –0.296 | 3.463 | 0.403 | <0.001 |
| Macular OCT signal strength | –0.039 | 0.510 | –0.103 | 5.625 | 0.002 | 0.410 |
| Average mGCIPL thickness | –0.553 | <0.001 | –0.145 | 15.415 | 0.296 | <0.001 |
| ONH OCT signal strength | –0.126 | 0.032 | –0.303 | 7.280 | 0.019 | 0.020 |
| Average pRNFL thickness | –0.597 | <0.001 | –0.102 | 12.896 | 0.338 | <0.001 |

<Modified sentence in result section>

“Age, sex, spherical equivalence, CCT, axial length, and macular OCT signal strength all showed no significant correlation with visual field prediction.”

**Comment 7)** Why did the authors use class activation maps in order to visualize important features? What about other explainability techniques (e.g. sensitivity analyses, occlusion or layer-wise relevance propagation)?

🡺 Thank you for comment. We think the XML techniques you recommended are good methods to see what happens inside the deep learning architecture. The reason why we used class activation map (CAM) was that 1) it was easy to apply, 2) the resulting heatmap image was well correlated with clinical studies. Please see the comparison below. Both images are generated from the same input OCT image. You can see that in each 52 CAM images (B), the activated region of ONH image (right side) is sectorally well correlated with visual field region (note that you can see orange and dark gray regions in structure-function relationship diagram (A) is well reflected in CAM image (B)). However, in layer-wise relevance propagation (LRP) map (C), the highly activated area (red colored) in ONH image does not fit to structure-function relationship and was difficult to interpret. This LRP images were generated using ‘innvestigate’ library (<https://github.com/albermax/innvestigate>).

(A) Regional structure-function relationship between combined OCT image (left) and visual field region (right). Regions of the same color are the areas that are related between structure and function. Macula scan is related to the dashed rectangular area in visual field diagram.

(B) Class Activation Map (CAM)

(C) Layer-wise relevance propagation (LRP)

**Comment 8)** I would recommend not using the term "artificial intelligence" (e.g., line 251, lines 363/364), but be more specific.

🡺 Thank you for comment. We modified “artificial intelligence” term like below.

<figure 3 legend (line 251)>

“the threshold values predicted by artificial intelligence are shown on the (C) right panel.” 🡪 “the threshold values predicted by Inception V3 based deep learning architecture are shown on the (C) right panel.”

<line 363/364>

“In Fig 4C, the CAM showed how artificial intelligence constructed this map” 🡪 “In Fig 4C, the CAM showed how Inception V3 based deep learning architecture constructed this map”

**Comment 9)** Line 66: Cite a few papers showing that deep learning performed comparable to humans.

🡺 Thank you for comment. We cited those papers below which showed a good performance as good as human doctor in fundus reading.

1) Peng, Y. et al. DeepSeeNet: A Deep Learning Model for Automated Classification of Patient-based Age-related Macular Degeneration Severity from Color Fundus

2) Burlina, P. et al. Utility of Deep Learning Methods for Referability Classification of Age-Related Macular Degeneration. JAMA Ophthalmol 136, 1305–1307 (2018).

3) Xu, Yi, et al. The diagnostic accuracy of an intelligent and automated fundus disease image assessment system with lesion quantitative function (SmartEye) in diabetic patients. BMC ophthalmology 19.1 (2019): 184.

**Comment 10)** Line 66: Machine learning? Especially deep learning allows for end-to-end learning. In classical machine learning, features are extracted beforehand.

🡺 Thank you for comment. You are absolutely right. This is our mistake to use term machine learning in the context. We modified “machine learning” to “deep learning algorithm” in line 66.

**Comment 11)** Line 92: Test dataset consists of 290 eyes of 290 subjects? Why only one eye per subject?

🡺 Thank you for comment. We included only one eye per subject. This is part of our efforts to evaluate more objectively. If one of the patient’s eye showed a good result, the other eye tends to show a good result too. We worried this can make the performance overestimated. One eye per one person method is statistically simple and may prevent such overestimation. Please understand our efforts to make it objectively.

**Comment 12)** Lines 409/410: Would the authors expect that results are not transferable to people from other ethnicities (just out of curiosity)? But it is a good point to make here!

🡺 Thank you for comment. In Girkin et al.’s study, “Variation in Optic Nerve and Macular Structure with Age and Race with Spectral-Domain Optical Coherence Tomography”, it was reported that optic nerve head (ONH), RNFL, and macular parameters measured by SD-OCT are all varied across racial groups. Because of this variation, they concluded that normal range must be differed in different populations. Thus, in different ethnics, the result may not be same as ours. As you recommended, we added some sentences describing Girkin’s study like below.

<newly added sentences (red color) in limitation paragraph>

“This study had a few limitations. First, only those patients with glaucoma were included to predict visual field. If patients with diseases other than glaucoma, where the visual field defect was not altitudinal in nature, such as temporal hemianopsia, were included, the prediction accuracy may be different than that reported in our study. Second, the training and test datasets were comprised of a primarily Korean population and the possibility of performance being different in populations comprising of other ethnicities should be considered. Girkin et al. [46] evaluated the effect of age and race on ONH, RNFL, and macular parameters obtained by SD-OCT. In their report, all parameters measured by SD-OCT were varied across racial groups. Thus, the visual field prediction from SD-OCT can be also varied across racial groups. Researchers must be aware of this.”

- The English in this document has been checked by at least two professional editors, both native speakers of English. For a certificate, please see: http://www.textcheck.com/certificate/GIKtlz
